# Basophils are dispensable for the establishment of protective adaptive immunity against primary and challenge infection with the intestinal helminth parasite *Strongyloides ratti*

**DOI:** 10.1371/journal.pntd.0006992

**Published:** 2018-11-29

**Authors:** Martina Reitz, Marie-Luise Brunn, David Voehringer, Minka Breloer

**Affiliations:** 1 Bernhard Nocht Institute for Tropical Medicine, Hamburg, Germany; 2 Department of Infection Biology, University Hospital Erlangen and Friedrich-Alexander University Erlangen-Nuernberg, Erlangen, Germany; Universidade Federal de Minas Gerais, BRAZIL

## Abstract

Infections with helminth parasites are controlled by a concerted action of innate and adaptive effector cells in the frame of a type 2 immune response. Basophils are innate effector cells that may also contribute to the initiation and amplification of adaptive immune responses. Here, we use constitutively basophil-deficient *Mcpt8-Cre* mice to analyze the impact of basophils during initiation and execution of the protective type 2 responses to both, a primary infection and a challenge infection of immune mice with the helminth parasite *Strongyloides ratti*. Basophil numbers expanded during parasite infection in blood and mesenteric lymph nodes. Basophil deficiency significantly elevated intestinal parasite numbers and fecal release of eggs and larvae during a primary infection. However, basophils were neither required for the initiation of a *S*. *ratti-*specific cellular and humoral type 2 immune response nor for the efficient protection against a challenge infection. Production of Th2 cytokines, IgG1 and IgE as well as mast cell activation were not reduced in basophil-deficient *Mcpt8-Cre* mice compared to basophil-competent *Mcpt8-WT* littermates. In addition, a challenge infection of immune basophil-deficient and WT mice resulted in a comparable reduction of tissue migrating larvae, parasites in the intestine and fecal release of eggs and L1 compared to mice infected for the first time. We have shown previously that *S*. *ratti* infection induced expansion of Foxp3^+^ regulatory T cells that interfered with efficient parasite expulsion. Here we show that depletion of regulatory T cells reduced intestinal parasite burden also in absence of basophils. Thus basophils were not targeted specifically by *S*. *ratti-*mediated immune evasive mechanisms. Our collective data rather suggests that basophils are non-redundant innate effector cells during murine *Strongyloides* infections that contribute to the early control of intestinal parasite burden.

## Author summary

Helminths are large multicellular parasites that infect approximately every third person. Infections are controlled by a concerted action of innate and adaptive immune responses. Basophils and mast cells are innate effector cells with overlapping functions that have recently been implicated in the initiation and promotion of adaptive immune responses. Here, we analyze the function of basophils during murine infection with *Strongyloides ratti*, an intestinal parasite with tissue migrating stages. Using mice constitutively lacking basophils, we show that their most important function is the early control of intestinal parasite burden during a primary infection. Basophils are not needed for efficient killing of tissue migrating *S*. *ratti* larvae, the timely termination of infection or the initiation and execution of protective immunity against *S*. *ratti* challenge infections in immune mice. Since basophils were shown to be important for the immunity against different helminth parasites in intestine or tissue (*Heligmosomoides polygyrus* and *Nippostrongylus brasiliensis)*, their function has to be evaluated for every parasite species individually.

## Introduction

Approximately one third of the human population is infected with parasitic helminths. *Strongyloides ratti* is a rodent-specific helminth parasite that can be used as a model parasite to study the immune response against intestinal helminth infections with tissue migrating stages in the mouse system [1]. Infective third stage larvae (L3i) actively penetrate the skin of the mammalian host, migrate within 2 days via the tissue and partially the lung towards the nasofrontal region of the host. They are swallowed by day 2–3 post infection (p.i.) and reach their final destination, the small intestine, where they molt twice to become parasitic female adults that live embedded in the mucosa of the intestine. The females reproduce via parthenogenesis by day 5–6 p.i. and release eggs and already hatched first stage larvae (L1) with the feces into the environment. Immune competent mice terminate the infection within a month and remain semi-resistant to subsequent infections [2, 3]. We have previously shown that the early expulsion of parasitic adults from the small intestine is predominantly mediated by mucosal mast cells [4]. Thereby, mast cells represented terminal effector cells that were not involved in killing of tissue-migrating L3 but were indispensable for the final expulsion of *S*. *ratti* during a primary infection. However, the generation of the adaptive immune response that mediates partial protection against a challenge infection of immune mice was readily established in the absence of mast cells.

Basophils are a rare population of late effector cells that are phenotypically and functionally related to mast cells. They arise from a common precursor and share a set of effector mediators that are stored in vesicles ready to be released [5]. In contrast to mast cells that differentiate and reside in the tissue, basophils leave the bone marrow as mature cells, are predominantly found in the blood stream and spleen and have a relatively short lifespan of about 60 h under steady-state conditions [6]. After exposure to stimuli such as allergens or parasitic helminth infections, basophils can be recruited to the site of inflammation. We and others have shown that, although clearly contributing to the early expulsion of *Strongyloides* adults from the intestine, basophils play only a minor role in the final control and termination of infection [4, 7]. Also intestinal parasite burden during a primary infection with *Nippostrongylus brasiliensis*, *Heligmosomoides polygyrus*, *Trichinella spiralis* and *Litomosoides sigmodontis* were not affected by basophil deficiency or depletion [8–11]. However, several lines of evidence suggest a contribution of basophils in the generation and execution of the protective immune response during a secondary infection with *N*. *brasiliensis* and *H*. *polygyrus* [6, 9, 10, 12, 13]. Thereby, possible functions of basophils in the polarization of Th2 immune responses, the early production of type 2 polarizing cytokines and antigen presentation are being debated [14, 15].

Here, we analyze the role of basophils in the protective immune response to a *S*. *ratti* challenge infection in immunized mice. Although basophils expanded during the course of infection, a protective adaptive immune response was readily established in the absence of basophils. Cytokine and antibody responses and subsequent mast cell activation were unchanged in basophil-deficient and basophil-competent mice. Efficient killing of migrating larvae as well as rapid expulsion of intestinal parasites during a challenge infection of immune mice was not impaired by basophil deficiency, thus ruling out a non-redundant role for basophils in the initiation or execution of a protective memory response to *S*. *ratti* infection.

## Methods

### Ethics and mice

Animal experiments were conducted in agreement with the German animal protection law and experimental protocols were approved by Federal Health Authorities of the State of Hamburg. BALB/c *Mcpt8-Cre* mice [10] and DEREG mice [16] have been described previously and were bred heterozygously. Wistar rats were obtained from Janvier Labs (Le Genest-Saint-Isle, France). Heterozygous BALB/c DEREG mice were intercrossed with heterozygous BALB/c *Mcpt8-Cre* mice in the animal facilities of the Bernhard Nocht Institute for Tropical Medicine to provide littermates for the experiments. All mice were bred in house and kept in individually ventilated cages under specific pathogen-free conditions. For all experiments, male and female mice were used at 7 to 10 weeks of age.

### *S*. *ratti* life cycle and infections

The *S*. *ratti* cycle was maintained in Wistar rats and infections were performed by s.c. infection of 2000 L3i in the hind footpad of mice [2]. Mice were vaccinated with 2000 irradiated L3i (160 Gy) 4 weeks before challenge infection with 2000 viable L3i as described [4]. For Treg depletion, groups of mice received 0.5 μg DT (Merck, Darmstadt, Germany) dissolved in PBS (pH 7.4) i.p. on three consecutive days, starting one day prior to *S*. *ratti* infection. Treg depletion was controlled by analysis of peripheral blood samples for GFP, Foxp3, and CD4 expression at day 1 p.i. Parasite burden in the intestine and quantification of the *S*. *ratti* 28S RNA-coding DNA in the feces of infected mice was performed as described [17, 18].

### Flow cytometry

For surface staining, cells were stained for 30 min on ice in the dark with FITC-labeled antibodies against CD4 (clone: RM4-5), CD8 (clone: 53–6.7) and CD19 cells (clone: 1D3), PerCP Cy5.5-labelled anti-mouse CD11b (clone: M1/70), PE-labelled anti-mouse IgE (clone: RME-1), Brilliant Violet 421-labelled anti-mouse CD117 (c-Kit; clone: 2B8) and PE Cy7-labelled anti-mouse CD49b (clone: DX5). Ab were purchased from BioLegend or Affymetrix eBioscience. For intracellular staining cells were permeabilized with 250 μl fixation/permeabilization buffer for 30 min at 4°C, washed with permeabilization buffer and stained with PE- or Alexa Fluor 700-labeled anti-Foxp3 (clone FJK-16s, eBiosciences, SanDiego, USA). Samples were analyzed on a LSRII Flow Cytometer (Becton Dickinson) using FlowJo software (TreeStar).

### Characterization of the cellular immune response

Mice were sacrificed either naïve or on day 6 p.i. A total of 2×10^5^ spleen and mesenteric lymph node (mLN) cells were cultured in 3–5 replicates 96-well round-bottom plates in RPMI 1640 medium supplemented with 10% FCS, 20 mM HEPES, L-glutamine (2 mM), and gentamicin (50 μg/mL) at 37°C and 5% CO_2_. The cells were stimulated for 72 h with *S*. *ratti* antigen lysate (20 μg/mL) or anti-mouse CD3 (145-2C11, 1 μg/mL) or with medium only. The supernatant was harvested for analysis of cytokine production by ELISA. IL-3, IL-4, IL-10 and IL-13 in culture supernatants were measured using DuoSet ELISA development kits (R&D Systems, Wiesbaden, Germany), according to the manufacturer’s instructions. IL-9 detection was performed by coating with 2 μg/mL anti-IL-9 Ab (BD, Heidelberg, Germany) overnight at 4°C. Plates were blocked with 10% FCS/0.05%Tween/PBS for 2 h at RT. Samples and recombinant IL-9 standard (Peprotech, Hamburg, Germany) were incubated overnight and detection was performed with an anti-IL-9-biotin AB (BD, Heidelberg, Germany) for 1 h at RT and subsequent Streptavidin-HRP incubation for 20 min before development with 100 μL/well tetramethylbenzidine 0.1 mg/ml, 0.003% H_2_O_2_ in 100 mM NaH_2_PO_4_ (pH 5.5). The reaction was stopped after 10 min by adding 25 μL of 2 M H_2_SO_4_.

### Characterization of the humoral immune response

Blood was collected from day 14 infected mice and *Strongyloides*-specific Ig in the serum was quantified by ELISA as described [2]. Briefly, 50 μL/well *S*. *ratti* Ag lysate (2.5 μg/mL) in PBS was coated overnight at 4°C on Microlon ELISA plates (Greiner, Frickenhausen, Germany). Plates were washed four times with PBS 0.05% Tween 20 and blocked by incubation with PBS 1% BSA for 2 h at RT. Serial dilutions of sera in PBS 0.1% BSA were incubated in duplicate, adding 50 μL/well overnight at 4°C. Plates were washed five times, and *Strongyloides*-specific Ig was detected by incubation with 50 μL/well horseradish peroxidase (HRP)-conjugated anti-mouse IgM, anti-mouse IgG1 or anti-mouse IgG2b (Zymed Karlsruhe, Germany) for 1 h at RT. Plates were washed five times and developed by incubation with 100 μL/well tetramethylbenzidine 0.1 mg/ml, 0.003% H_2_O_2_ in 100 mM NaH_2_PO_4_ (pH 5.5) for 2.5 min. Reaction was stopped by addition of 25 μL/well 2 M H_2_SO_4_, and OD at 450 nm (OD_450_) was measured. The titer was defined as the highest dilution of serum that led to an OD_450_ above the doubled background. Background was always below 0.15 OD_450_. Concentration of IgE was quantified using the IgE ELISA kit (BD, Heidelberg Germany) according to the manufacturers recommendations.

### Mast cell activity

For analysis mouse mast cell protease-1 (mMCPT-1), blood was collected from infected mice at the indicated time points and allowed to coagulate for 1 h at room temperature (RT). Serum was collected after centrifugation at 10,000× *g* for 10 min at RT and mMCPT-1was detected using the mMCPT-1 ELISA Ready-SET-Go kit (eBioscience, San Diego, USA) according to the manufacturers recommendations.

### Statistical analysis

All data were assessed for normality. Groups were compared by using Mann Whitney-U test (non-parametric comparison of two groups) or Kruskal-Wallis test corrected with Dunn’s multiple comparisons test (non-parametric multiple comparisons) or 2 Way ANOVA with Bonferroni post test (parametric comparison of two groups over time), using GraphPad Prism software (San Diego). P values of ≤0.05 were considered to indicate statistical significance.

## Results

### Basophils expand during *S*. *ratti* infection

**Fig 1:**
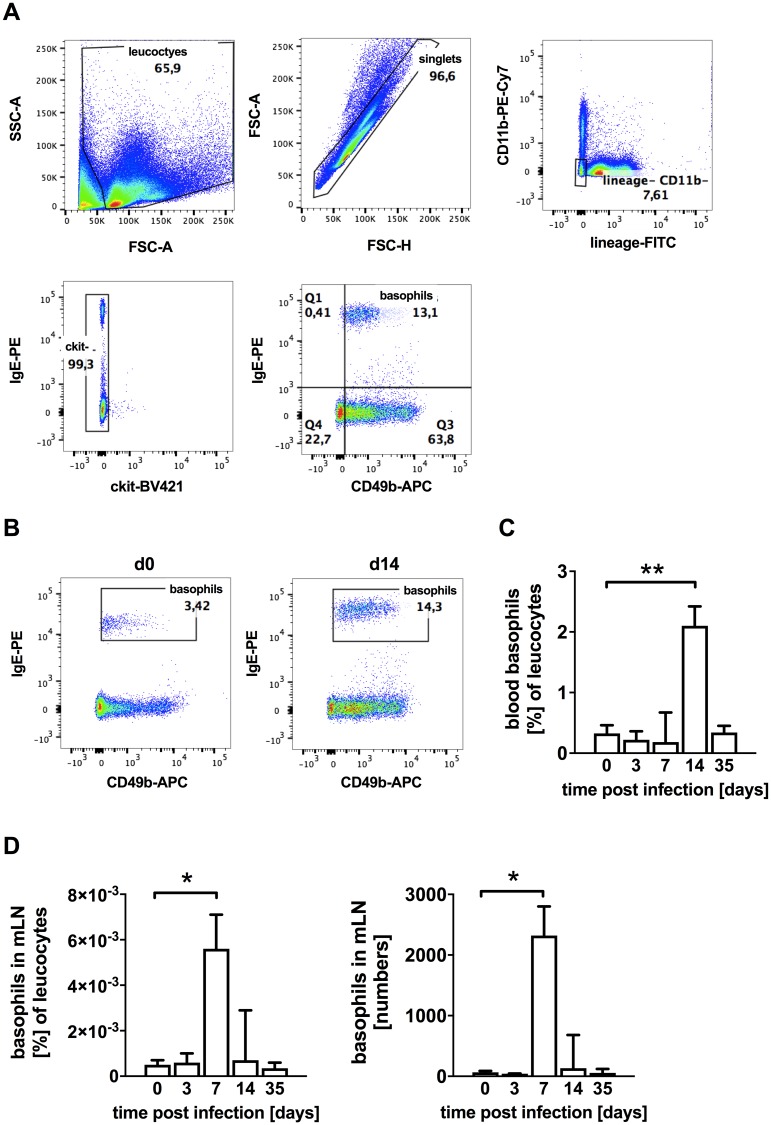
Basophil expansion in *S*. *ratti* infected BALB/c mice. BALB/c mice were infected with 2000 L3i *S*. *ratti* s.c. into the hind footpad. Blood and mesenteric lymph node (mLN) cells were collected from each mouse at the indicated time points after *S*. *ratti* infection and basophils were stained as lineage (CD4, CD8, CD19, CD11b), c-kit (CD127) negative and IgE and CD49b positive cells and analyzed by flow cytometry (**A)** Gating strategy (**B**) Representative dot plots of blood basophils comparing day 0 and day 14 (**C**) Percentage of blood basophils (**D**) Percentage and absolute numbers of mLN basophils. Graphs in **(C)** and **(D)** show the median with 95% CI (confident interval) of two independent experiments (n = 7–8 per time point and group). *P<0.05: the infected groups differed significantly to naïve groups (day 0) as determined by Kruskal-Wallis test corrected with Dunn’s multiple comparisons test.

To analyze a potential role of basophils during *S*. *ratti* infection we first monitored their expansion in the blood and mLN by flow cytometry (Fig 1). Basophil expansion in the skin was not quantified in this study. Basophils were identified as lineage negative (i.e. CD19^-^, CD4^-^, CD8^-^), CD11b negative, and c-kit negative cells that were positive for IgE and CD49b [6] (Fig 1A). Blood basophil numbers remained at baseline levels at day 3 p.i., a time point that marks the end of the L3 tissue migration phase and at day 7 p.i. that is the peak of parasite burden in the intestine. Blood basophil numbers increased one week after the peak of intestinal parasite burden, at day 14 p.i., returned to naïve levels at the resolution of infection by day 35 p.i. (Fig 1B and 1C). In the mLN, basophils increased in frequency and numbers already at day 7 p.i., the time point of maximal worm burden, and decreased again to show a non-significant trend towards elevation at day 14 p.i followed by contraction to baseline level at clearance of infection at day 35 p.i. (Fig 1D). Thus, *S*. *ratti* infection resulted in expansion of basophils both systemically in the blood and locally in the mLN with kinetics that correlated to the presence of parasitic *S*. *ratti* adults in the intestine.

### Intact *S*. *ratti*-specific immune response is established in basophil-deficient mice

**Fig 2:**
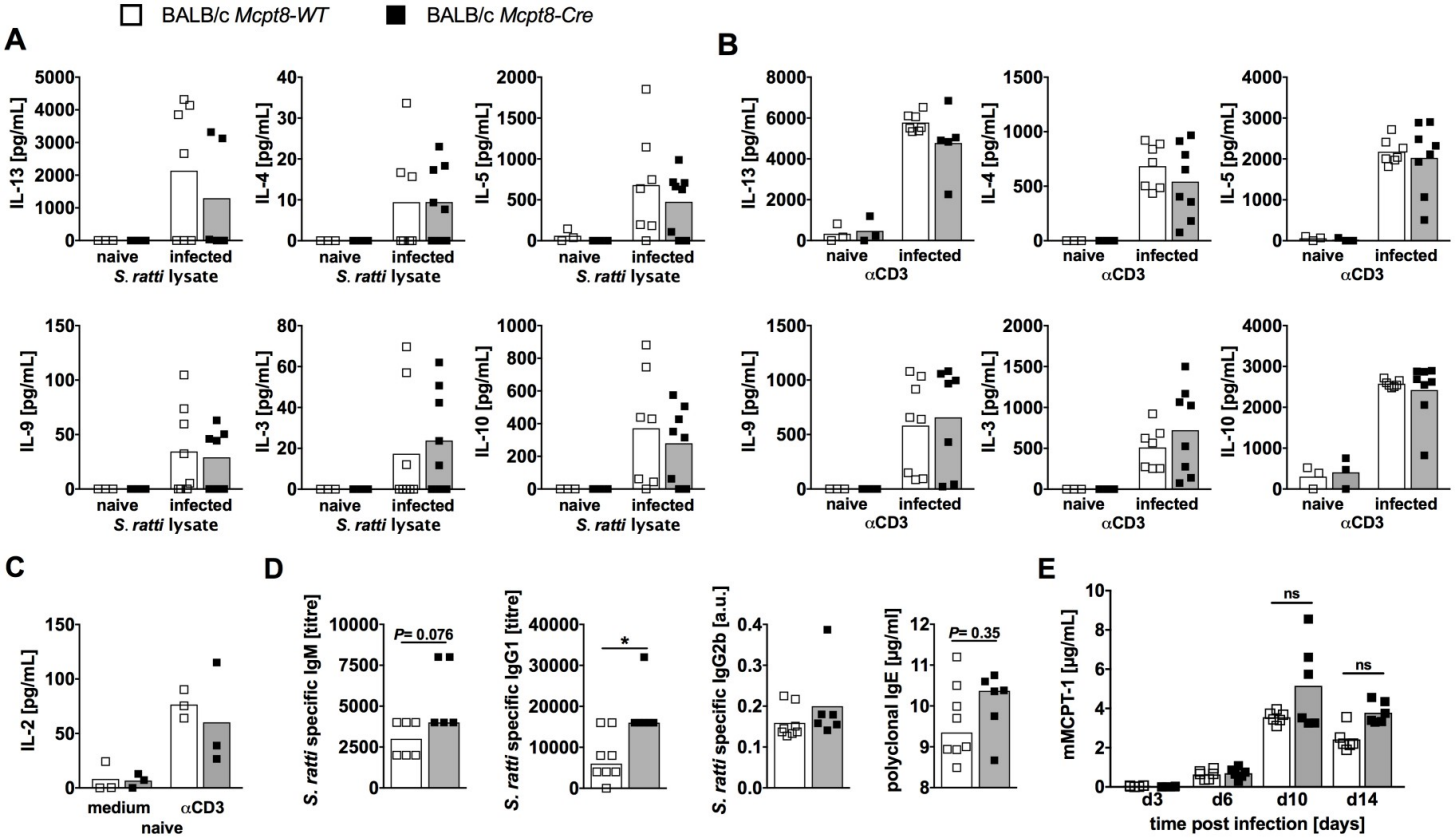
Immune response in *S*. *ratti*-infected basophil-deficient *Mcpt8-Cre* mice. Basophil-deficient BALB/c *Mcpt8-Cre* mice (black squares) and basophil-competent littermates BALB/c *Mcpt8-WT* (open squares) were infected with 2000 L3i *S*. *ratti* s.c. into the hind footpad. Cytokine production by *S*. *ratti* antigen lysate (20 μg/mL) (**A**) or α-CD3 (1 μg/mL) (**B**) stimulated mLN cells derived from naïve or from day 6 infected mice was quantified by ELISA (n = 3–9). **(C)** IL-2 production by mLN cells derived from naïve mice cultured in medium or in the presence of αCD3 (1 μg/mL) was compared. **(D)** *S*. *ratti*-specific IgM, IgG1, IgG2b titers and IgE concentration in sera taken from day 14 infected mice were measured by ELISA (n = 6–9). (**E)** mMCPT-1 serum concentration in infected mice was quantified by ELISA at the indicated time points (n = 7). Graphs show combined data from two independent experiments, naïve mice were only analyzed once. Each symbol represents an individual mouse and bars indicate the median **(A-D)** or mean **(E)**. * *P*<0.05: *Mcpt8-Cre* and *Mcpt8-WT* groups differed significantly, as determined by Mann-Whitney *U* test **(A-D)** or 2 Way ANOVA with Bonferroni post test **(E)**.

To analyze the impact of the expanding basophil population on the generation of the anti-helminth immune response, we compared the T and B cell responses in constitutively basophil-deficient *Mcpt8-Cre* mice and basophil-competent *Mcpt8-WT* mice (Fig 2).

Thereby the absence of basophils in basophil-deficient *Mcpt8-Cre* during the entire course of *S*. *ratti* infection was controlled by quantification of basophils as CD19, CD4, CD8, CD11b, c-kit negative and IgE, CD49b positive cells in the peripheral blood of *S*. *ratti-*infected *Mcpt8-Cre* and *Mcpt8-WT* littermates (S1 Fig).

*Ex vivo* re-stimulation of mLN cells derived from day 6 *S*. *ratti* infected mice with crude *S*. *ratti* antigen (Fig 2A) or anti-CD3 mAb (Fig 2B) elicited secretion of Th2 associated cytokines such as IL-13, IL-4 and IL-5 in basophil-deficient and basophil-competent mice to the same extent. Production of IL-9 and IL-3, cytokines that are central in immunity to *Strongyloides* [19, 20], were unchanged by basophil deficiency. Finally, the production of IL-10 that may, according to context and parasite species, contribute to protection or immune evasion [21] was not modulated by basophil deficiency. The observed cytokine production was infection-specific as mLN cells derived from naïve *Mcpt8-WT* and *Mcpt8-Cre* mice did not respond to *S*. *ratti* antigen-specific stimulation. Naïve mLN cells produced very low amounts of IL-13 and IL-10 and failed to produce IL-4, IL-5, IL-9 or IL-3 in response to polyclonal anti-CD3-mediated stimulation reflecting the expected absence of immune activation or polarization (Fig 2B). However, naïve mLN produced IL-2 in response to anti-CD3 stimulation, thus proving their general viability (Fig 2C). Also spleen cells derived from day 6 *S*. *ratti*-infected *Mcpt8-WT* and *Mcpt8-Cre* mice displayed comparable cytokine production to either CD3-engagement or *S*. *ratti* antigen-specific stimulation while unstimulated spleen cells did not secrete cytokines (S2 Fig). Absence of basophils did not impair the antibody response at day 14 p.i. (Fig 2D). The serum concentration of *S*. *ratti-*specific IgG2b was unchanged in basophil deficient *Mcpt8-Cre* mice. Antigen-specific IgE was not detectable in complete or in IgG1-depleted sera of *S*. *ratti-*infected mice (S3 Fig) but polyclonal IgE concentrations were not reduced in basophil deficient mice. Interestingly, serum concentrations of *S*. *ratti-*specific IgM and IgG1, the isotypes that predominantly mediate opsonization and subsequent clearance of migrating L3 in the tissues [22, 23], were even increased by trend (IgM) or statistically significant (IgG1) in basophil-deficient *Mcpt8-Cre* mice compared to their basophil-competent *Mcpt8-WT* littermates (Fig 2D).

Final expulsion of *S*. *ratti* adults from the small intestine depends on activated mucosal mast cells [4]. We measured mast cell activation by quantification of mouse mast cell protease 1 (mMCPT-1) that is specifically released by degranulating mucosal mast cells [24]. The concentration of mMCPT-1 in the serum was unchanged in basophil-deficient *Mcpt8-Cre* mice and their basophil-competent littermates from day 3 to day 14 p.i (Fig 2E), indicating comparable mast cell activation in the presence and absence of basophils.

In summary, we did not record a dominant impact of basophils on the initiation of helminth-specific Th2 and Th9 response.

### Efficient protection against primary and challenge *S*. *ratti* infections in basophil-deficient mice

**Fig 3:**
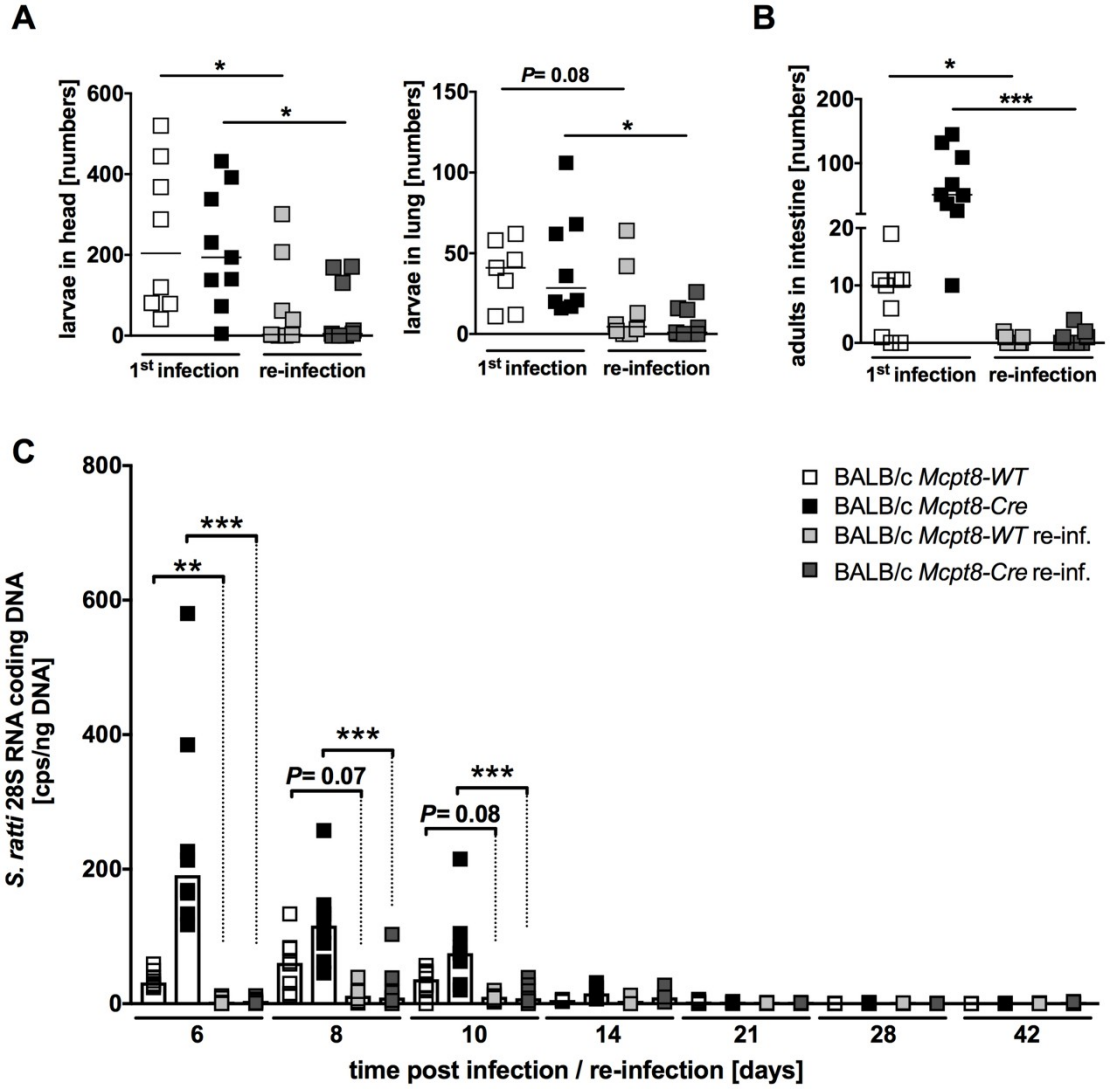
*S*. *ratti* challenge infection in basophil-deficient *Mcpt8-Cre* mice. BALB/c *Mcpt8-Cre* mice (dark squares/bars) and their respective littermates BALB/c *Mcpt8-WT* (light squares/bars) were s.c. infected with 2000 *S*. *ratti* L3i into the hind footpad. (**A)** Number of migrating larvae on day 2 p.i. in head and lung (n = 7–9). (**B)** Number of parasitic adults in the intestine on day 6 p.i. (n = 7–10). (**C)** Release of *S*. *ratti* 28S RNA-coding DNA in the feces at the indicated time points, as measured by qPCR (n = 8). Graphs show combined data of two independent experiments, each symbol represents an individual mouse, lines **(A,B)** or bars **(C)** indicate the median. *P<0.05; **P<0.01; ***P<0.001: significant difference between primary infection and challenge infection, as determined by Kruskal-Wallis test with Dunn’s multiple comparisons. For the kinetic shown in **(C)** each time point was analyzed separately.

Immune competent mice terminate *Strongyloides* infection within a month and remain semi-resistant to a secondary infection [22, 25]. Thereby already migrating L3 in the tissues of immune mice are potently attacked and killed and only few parasitic adults can be detected in the small intestine of immune mice. We have shown previously that vaccination with irradiated *S*. *ratti* L3i that cannot molt to parasitic adults confers the same immunity to a challenge infection as a resolved patent primary infection [4]. To evaluate the impact of basophils on protection of immune mice during challenge infection, basophil-deficient and basophil-competent mice were vaccinated with irradiated *S*. *ratti* L3 and 4 weeks later challenge infected with viable L3. Migrating L3 numbers in the tissue (head and lung) were recorded on day 2 post re-infection and parasitic adults were counted at day 6 post re-infection in the small intestine and compared to age- and gender-matched non-vaccinated mice that were infected for the first time (Fig 3). To additionally monitor the kinetic of infection we measured *S*. *ratti*-derived DNA in the feces as a rough indicator for release of eggs and first stage L1 [2]. Regarding the primary infection, basophil deficiency did not change the number of L3 in the head or lung (Fig 3A) but elevated intestinal parasite burden (Fig 3B) and fecal output of *S*. *ratti*-derived DNA (Fig 3C) as we had shown before [4]. Although intestinal parasite burden and L1 release were elevated in basophil-deficient mice during a primary infection, the kinetic of infection termination was not changed as all mice cleared the infection by day 28 p.i. (Fig 3C). Both, vaccinated basophil-deficient *Mcpt8-Cre* mice and vaccinated basophil-competent *Mcpt8-WT* mice showed drastically reduced L3 numbers in the head during challenge infection compared to their naïve counterparts that were *S*. *ratti* infected for the first time (Fig 3A). Thus, protection against the tissue migrating L3 was established in the absence of basophils. Furthermore, almost no parasitic adults were detectable in day 6 challenge infected mice compared to mice that were infected for the first time irrespective of the presence or absence of basophils (Fig 3B). The reduced parasite burden in the small intestine during challenge infection was reflected by a reduced release of *S*. *ratti* DNA in the feces of vaccinated *Mcpt8-WT* and *Mcpt8-Cre* mice from day 6 to day 14 post re-infection (Fig 3C). The intact immunity in *Mcpt8-Cre* mice was not caused by a repopulation of basophils during primary infection or challenge infection (S1 Fig).

In summary, these results show that basophils contribute to the control of intestinal parasite burden during a primary infection but are dispensable for the protection during challenge infection of immune mice regarding both, the efficient control of tissue migrating L3 and the expulsion of parasitic adults from the small intestine.

### *S*. *ratti*-induced and Treg-mediated immune evasion does not target basophils

**Fig 4:**
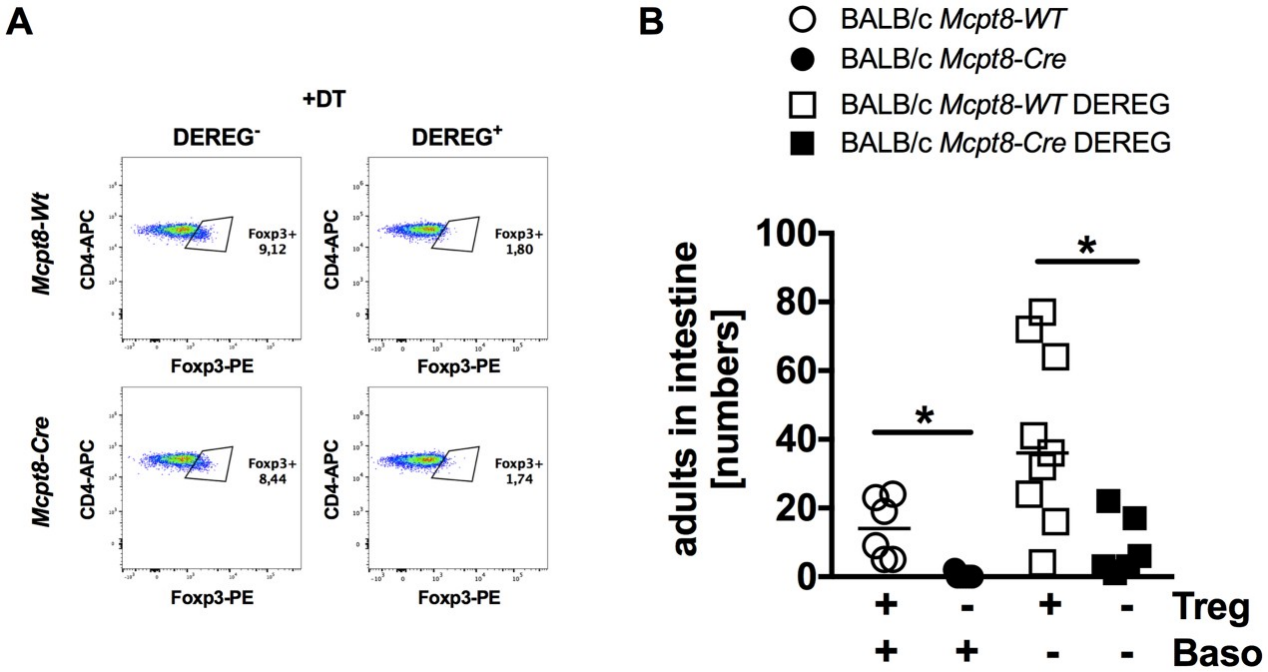
Treg depletion in basophil-deficient *Mcpt8-Cre* mice. BALB/c *Mcpt8-WT* (open circles), BALB/c DEREG (closed circles), BALB/c *Mcpt8-Cre* (open squares) and BALB/c *Mcpt8-Cre* DEREG (closed squares) mice were treated with DT on three consecutive days starting one day before s.c infection with 2000 *S*. *ratti* L3i. (**A)** Depletion of Tregs was controlled by flow cytometry on day 1 p.i. by intracellular staining of Foxp3. (**B)** Numbers of parasitic adults in the small intestine on day 6 p.i. Graphs show combined data from two independent experiments (n = 6–9), lines indicate the median. *P<0.05; ***<0.0001: Parasite burden in DEREG positive Treg-depleted and DEREG negative undepleted groups differed significantly, as determined by Kruskal-Wallis test corrected with Dunn’s multiple comparisons.

The combined results of this study rule out a central role for basophils in the generation and execution of the adaptive anti-helminth response during a secondary infection. However, within this study we reproduced our earlier results showing a non-redundant contribution of basophils, next to mast cells, in the expulsion of *S*. *ratti* parasitic adults from the small intestine during a primary infection ([4] and Fig 3). We have shown previously that mast cell activation and subsequent parasite expulsion was suppressed by Foxp3^+^ regulatory T cells (Treg) that expanded upon *S*. *ratti* infection [18, 26]. Since mast cells and basophils are innate effector cells with overlapping functions that both contribute to the early control of intestinal *S*. *ratti* burden, we asked, whether Treg would also directly interfere with basophil function during *S*. *ratti* infection. To this end we crossed basophil-deficient *Mcpt8-Cre* mice to Depletion of Treg (DEREG) mice. DEREG mice are transgenic for a bacterial artificial chromosome driving the expression of a fusion protein consisting of the diphtheria toxin receptor (DTR) and enhanced green fluorescent protein (eGFP) under the control of the Foxp3 promoter [16]. The application of diphtheria toxin (DT) leads to a rapid and transient depletion of Foxp3^+^ regulatory T cells in mice that are heterozygous for the DEREG allele. Already the heterozygous expression of the Cre recombinase under the control of the Mcpt8 promoter in *Mcpt8-Cre* mice is sufficient to induce constitutive and complete depletion of basophils. Therefore the F1 of *Mcpt8-Cre* and DEREG mice yielded four different genotypes and phenotypes: basophil-competent *Mcpt8-WT* mice and basophil-deficient *Mcpt8-Cre* mice that were both DEREG negative and thus not susceptible to DT-mediated Treg depletion as well as basophil-competent *Mcpt8-WT* DEREG mice and basophil-deficient *Mcpt8-Cre* DEREG mice that were both susceptible to DT-mediated Treg depletion. All groups were treated with DT one day before *S*. *ratti* infection and the frequency of Treg in the peripheral blood was measured day 1 p.i. to control efficient Treg depletion in DEREG positive groups compared to DEREG negative littermates (Fig 4A). Parasite burden in the small intestine was counted at day 6 p.i. (Fig 4B). Depletion of Treg in basophil-competent BALB/c DEREG mice reduced parasite burden in the intestine as we have shown before [18, 26], demonstrating that Foxp3^+^ Treg interfered with efficient parasite expulsion. Basophil deficiency elevated the parasite burden in the presence of normal Treg frequency as observed before (Fig 3B). However, depletion of Treg in basophil-deficient mice still led to a significant reduction of this initially higher intestinal parasite burden. Thus, Treg interfered with efficient expulsion of *S*. *ratti* independently of the presence and thus independent of the function of basophils.

## Discussion

Analyzing the role of basophils during intestinal helminth infection of mice we demonstrate that basophils expanded during infection with *S*. *ratti* and specifically contributed to the early control of intestinal parasite burden. Basophils were not involved in the control of *S*. *ratti* tissue migrating larvae during primary infection and were dispensable for the generation of a protective Th2/9 immune response during *S*. *ratti* infection. Parasite-specific cytokine and Ab production as well as mast cell activation were not reduced in *S*. *ratti*-infected *Mcpt8-WT* and *Mcpt8-Cre* mice. Moreover, numbers of migrating larvae in the tissue, numbers of parasitic adults in the intestine and fecal release of eggs and L1 in a secondary infection were not affected by basophil deficiency.

In line with our results, IgG1 and IgE production, eosinophilia and Th2 expansion were not affected by constitutive basophil deficiency in *Mcpt8-Cre* mice during primary *H*. *polygyrus* and *N*. *brasiliensis* infection [9, 10]. Basophils were also not required for the initiation of a Th2 response to *Schistosoma mansoni* in constitutive basophil-deficient Basoph8 x Rosa-DTa mice [27] or *Mcpt8-Cre* mice [28]. Moreover, *Litomosoides sigmodontis* infection, a model for human filarial infections [29] elicited comparable cellular and humoral immune responses and was cleared with similar kinetics in basophil-deficient *Mcpt8-Cre* mice and *Mcpt8-WT* mice [11].

By contrast, depletion of basophils by injection of diphtheria toxin to BaS-DTR mice during *T*. *spiralis* infection led to an impaired production of type 2 associated cytokines such as IL-5, IL-4 IL-13 [8]. However, as the diminished Th2 cytokine production had no impact on intestinal parasite burden in the basophil-depleted *T*. *spiralis*-infected mice, the clinical relevance of the observed alteration in cytokine production appeared to be limited. In this context, also we cannot formally rule out a contribution of basophils to the amplification of the anti-*S*. *ratti* Th2/9 response after day 6 p.i. (i.e. the time point analyzed here) that had no impact on the kinetics of parasite clearance or parasite burden during a challenge infection.

One hallmark of effective initiation of an adaptive immune response to parasite infection is the establishment of a potent immune memory that is characterized by a more efficient protection against a secondary infection in many models of parasite infection. Our results show that the established anti-*S*. *ratti* Th2/9 immune response in the absence of basophils was readily translated into protection against a secondary challenge infection where hardly any adult parasite was detected in the small intestine. In line with this finding, other mouse models for basophil deficiency, basophil depleted MasTRECk and Mcpt8^DTR^ mice, were protected against a secondary infection with the closely related helminth *S*. *venezuelensis* [7].

By contrast, absence of basophils clearly impaired the efficient control of parasite burden during a secondary infection with either *N*. *brasiliensis* or *H*. *polygyrus*, although via different mechanisms [9]. Using mixed bone marrow chimeras, the authors showed that specifically the absence of basophil-derived IL-4 and IL-13 resulted in reduced Th2 cell expansion and increased intestinal parasite burden during secondary *H*. *polygyrus* infection [9]. Interestingly Th2 cell expansion and IgE response were intact during secondary *N*. *brasiliensis* infection in basophil-deficient mice [9, 10]. Here, basophils were important as effector cells mediating efficient eradication of migrating *N*. *brasiliensis* larvae already in the skin [12]. Comparable to *S*. *ratti*, *N*. *brasiliensis* larvae penetrate the skin and migrate within 3–4 days via the lung and mouth to the small intestine. Depletion of basophils by DT injection into Mcpt8^DTR^ mice before a secondary *N*. *brasiliensis* infection abolished larval retention in the skin and caused a higher parasite burden in the lung. Thereby, larval trapping in the skin depended on IgE and FcεRI-mediated activation of basophils [12]. A similar role for-IgE activated basophils was demonstrated in mediating resistance to a secondary tick infection [30].

Although immunity to secondary *S*. *ratti* infections is also characterized by efficient killing of tissue migrating larvae [3], attack and trapping of *Strongyloides* larvae predominantly depends on eosinophils and neutrophils that are activated by complement and IgG [22, 31–35]. In line with these reports we did not record changed numbers of tissue migrating larvae in basophil-deficient mice neither during primary nor challenge infection, suggesting that basophils are not as central for trapping of tissue migrating *Strongyloides* larvae as for trapping of *N*. *brasiliensis* larvae [12].

In summary, our results show that the dominant function of basophils during *S*. *ratti* infection in mice is the promotion of early intestinal parasite expulsion that is mediated by both basophils and mast cells [4, 7]. Although basophil-deficient mice displayed a higher intestinal parasite burden at day 6 p.i., clearance of infection was executed with WT kinetics within 4 weeks showing that lack of basophils as intestinal effector cells can be compensated by other cells. In sharp contrast, deficiency of mast cells resulted in prolonged infection to more than 20 weeks pointing out the non-redundant function of IL-9 activated mast cells as intestinal effectors during *S*. *ratti* infection [4, 20].

Although *S*. *ratti* infection is terminated after 4 weeks by immune competent mice, we have shown before that, in order to establish intestinal infection and to survive this 4 weeks, *S*. *ratti* actively down-modulates the immune response that promotes its expulsion [18, 26, 36]. *S*. *ratti* infection induces expansion of Treg and their depletion drastically reduced intestinal parasite burden in BALB/c mice [18, 26]. Since additional mast cell deficiency abrogated the beneficial effect of Treg depletion, the expanding Treg population directly interfered with mast cell mediated parasite expulsion [18]. In the current study, we show that basophil-deficiency did not abrogate the beneficial effect of Treg depletion. Although the basophil-deficiency alone elevated intestinal *S*. *ratti* parasite burden compared to basophil-competent mice, additional Treg depletion significantly reduced the intestinal parasite burden in the absence of basophils. Thus, basophil function in the intestine is not specifically suppressed by *S*. *ratti-*mediated immune evasive mechanism, emphasizing their minor role in the efficient control of this particular parasite.

## Supporting information

S1 Fig Blood basophils in *Mcpt8-Cre* and *Mcpt8-Wt* mice during primary *S*. *ratti* infection and challenge infection. (PDF)

S2 Fig Cytokine production of *ex vivo* stimulated spleen cells derived from *S*. *ratti*-infected basophil-deficient *Mcpt8-Cre* and basophil-competent *Mcpt8-WT* mice. (PDF)

S3 Fig Quantification of *S*. *ratti* specific IgE in IgG depleted mouse sera of *S*. *ratti* infected *Mcpt8-Wt* and *Mcpt8-Cre* mice. (PDF)
