# Supplementary material for: Basophils are dispensable for the establishment of protective adaptive immunity against primary and challenge infection with the intestinal helminth parasite Strongyloides ratti
Source: PLoS Negl Trop Dis. 2018 Nov 29;12(11):e0006992. doi: 10.1371/journal.pntd.0006992 (PMC6289456; doi:10.1371/journal.pntd.0006992)
Supplement: S1 Fig — (PDF) [file pntd.0006992.s001.pdf]

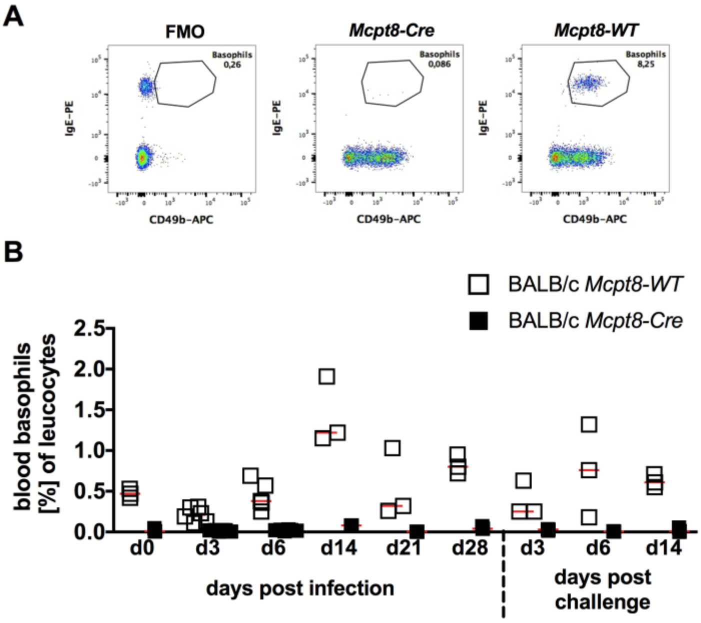

S1 Fig. Blood basophils in *Mcpt8-Cre* and *Mcpt8-Wt* mice during primary *S. ratti* infection and challenge infection. Basophil-deficient BALB/c *Mcpt8-Cre* mice (black squares) and basophil-competent BALB/c *Mcpt8-WT* littermates (open squares) were infected with 2000 L3i *S. ratti* s.c. into the hind footpad and again challenge infected with 2000 L3i *S. ratti* after 35 days. Basophils were stained as lineage (CD4, CD8, CD19, CD11b), c-kit (CD127) negative and IgE and CD49b positive cells and analyzed by flow cytometry. (**A**) Representative dot plots of blood basophils in BALB/c *Mcpt8-Cre* and BALB/c *Mcpt8-WT* mice on day 14 post infection. FMO (fluorescence minus one) shows staining without CD49b-APC antibody. (**B**) Percent of blood basophils in leucocytes during primary and secondary infection (n = 3-9), lines show median.
