# Supplementary material for: Basophils are dispensable for the establishment of protective adaptive immunity against primary and challenge infection with the intestinal helminth parasite Strongyloides ratti
Source: PLoS Negl Trop Dis. 2018 Nov 29;12(11):e0006992. doi: 10.1371/journal.pntd.0006992 (PMC6289456; doi:10.1371/journal.pntd.0006992)
Supplement: S2 Fig — (PDF) [file pntd.0006992.s002.pdf]

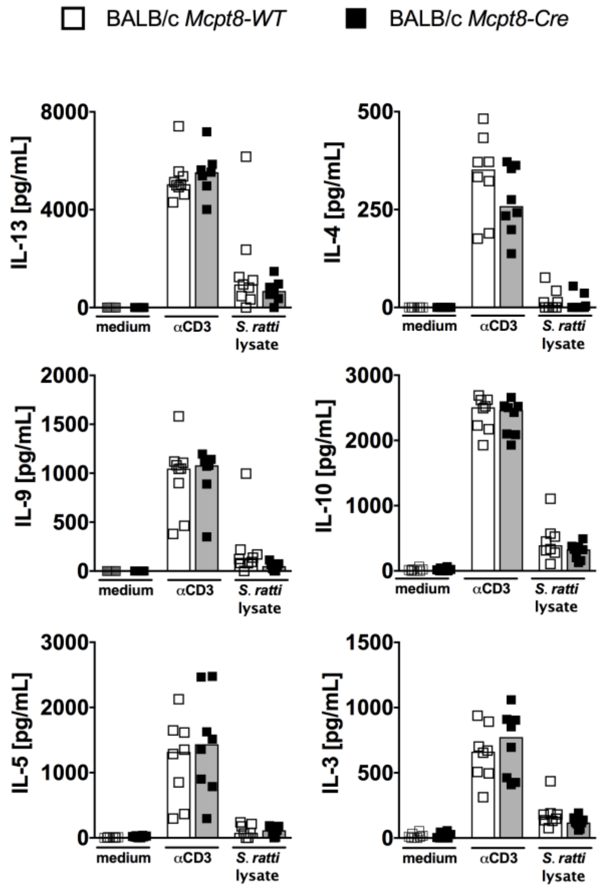

S2 Fig. Cytokine production of *ex vivo* stimulated spleen cells derived from *S. ratti*-infected basophil-deficient *Mcpt8-Cre* and basophil-competent *Mcpt8-WT* mice. Basophil-deficient BALB/c *Mcpt8-Cre* mice (black squares) and basophil-competent littermates BALB/c *Mcpt8-WT* (open squares) were infected with 2000 L3i *S. ratti* s.c. into the hind footpad. Cytokine production by *S. ratti* lysate (20 µg/mL) or αCD3 (1µg/mL) activated or in medium incubated spleen cells derived from day 6 infected mice was quantified by ELISA. Shown are combined data from two independent experiments, each symbol represents an individual mouse and bars indicate median (n = 6-9). No significant changes were recorded between basophil-deficient BALB/c *Mcpt8-Cre* mice and basophil-competent BALB/c *Mcpt8-WT* littermates as determined by Kruskal-Wallis test with Dunn's multiple comparison for each type of stimulation.
