# Supplementary material for: Basophils are dispensable for the establishment of protective adaptive immunity against primary and challenge infection with the intestinal helminth parasite Strongyloides ratti
Source: PLoS Negl Trop Dis. 2018 Nov 29;12(11):e0006992. doi: 10.1371/journal.pntd.0006992 (PMC6289456; doi:10.1371/journal.pntd.0006992)
Supplement: S3 Fig — (PDF) [file pntd.0006992.s003.pdf]

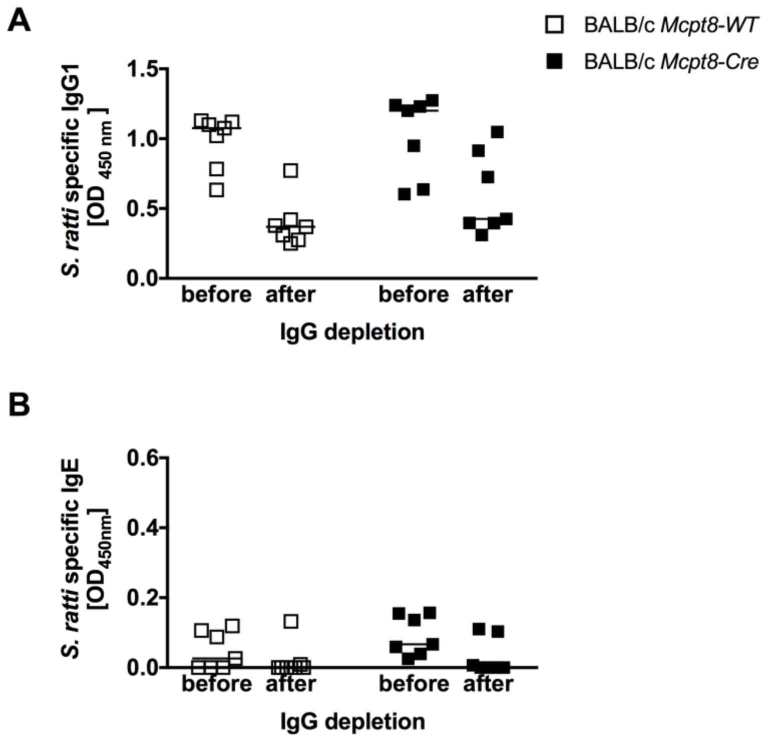

S3 Fig . Quantification of *S. ratti* specific IgE in IgG depleted mouse sera of *S. ratti* infected *Mcpt8-Wt* and *Mcpt8-Cre* mice. Basophil-deficient BALB/c *Mcpt8-Cre* mice (black circles) and basophil-competent littermates BALB/c *Mcpt8-WT* (open circles) were infected with 2000 L3i *S. ratti* s.c. into the hind footpad and serum was taken on day 21 and 28 post infection. Sera from both time points was pooled and IgG was depleted using Protein G HP SpinTrap / Ab Spin Trap columns (GE Healthcare). *S. ratti* specific IgG1 and IgE were measured by ELISA in a serum dilution of 1:100 (**A**) S. ratti specific IgG1 in sera before and after IgG depletion. (**B**) *S. ratti* specific IgE in sera before and after IgG depletion. Each symbol represents pooled sera from an individual mouse (n= 7), lines show the median.
